# Modeling cancer immunoediting in tumor microenvironment with system characterization through the ising-model Hamiltonian

**DOI:** 10.1186/s12859-022-04731-w

**Published:** 2022-05-30

**Authors:** Alfonso Rojas-Domínguez, Renato Arroyo-Duarte, Fernando Rincón-Vieyra, Matías Alvarado-Mentado

**Affiliations:** 1 Postgraduate Studies and Research Division, Tecnológico Nacional de México – IT de León, León, Mexico; 2 Depto. de Física, CUCEI, Universidad de Guadalajara, Guadalajara, Mexico; 3 Depto. de Computación, CINVESTAV-IPN, Av. Instituto Politécnico Nacional 2508, Col. San Pedro Zacatenco, GAM, 07360 Mexico City, CDMX Mexico

**Keywords:** Tumor immune interaction, Cancer micro-environment, CI emergent behavior, Immunoediting, Ising energy function

## Abstract

**Background and objective:**

Cancer Immunoediting (CI) describes the cellular-level interaction between tumor cells and the Immune System (IS) that takes place in the Tumor Micro-Environment (TME). CI is a highly dynamic and complex process comprising three distinct phases (Elimination, Equilibrium and Escape) wherein the IS can both protect against cancer development as well as, over time, promote the appearance of tumors with reduced immunogenicity. Herein we present an agent-based model for the simulation of CI in the TME, with the objective of promoting the understanding of this process.

**Methods:**

Our model includes agents for tumor cells and for elements of the IS. The actions of these agents are governed by probabilistic rules, and agent recruitment (including cancer growth) is modeled via logistic functions. The system is formalized as an analogue of the Ising model from statistical mechanics to facilitate its analysis. The model was implemented in the Netlogo modeling environment and simulations were performed to verify, illustrate and characterize its operation.

**Results:**

A main result from our simulations is the generation of emergent behavior *in silico* that is very difficult to observe directly *in vivo* or even *in vitro*. Our model is capable of generating the three phases of CI; it requires only a couple of control parameters and is robust to these. We demonstrate how our simulated system can be characterized through the Ising-model energy function, or Hamiltonian, which captures the “energy” involved in the interaction between agents and presents it in clear and distinct patterns for the different phases of CI.

**Conclusions:**

The presented model is very flexible and robust, captures well the behaviors of the target system and can be easily extended to incorporate more variables such as those pertaining to different anti-cancer therapies. System characterization via the Ising-model Hamiltonian is a novel and powerful tool for a better understanding of CI and the development of more effective treatments. Since data of CI at the cellular level is very hard to procure, our hope is that tools such as this may be adopted to shed light on CI and related developing theories.

**Supplementary Information:**

The online version contains supplementary material available at 10.1186/s12859-022-04731-w.

## Introduction

The scientific understanding of the interaction between the Immune System (IS) and cancer has come a long way since the formal postulation of the cancer immunosurveillance hypothesis by Burnet & Thomas in the late 1950s. Said development, however, has not been without difficulties due to both technological limitations as well as justified resistance from the scientific community to embrace certain ideas that were only partially supported by the evidence available at the time. By the beginning of the twenty-first century, several important discoveries had guided the evolution of the immunosurveillance hypothesis into the concept of cancer immunoediting (CI); a brief review of that evolution is found in [1, 2]. Two decades later, CI is a well established idea describing the process whereby the IS can both protect against cancer development as well as, over time, promote the appearance of tumors with reduced immunogenicity [3].

Cancer immunoediting comprises three phases that take place in a Tumor Micro-Environment (TME): (1) Elimination (immunosurveillance), wherein the IS detects and largely eliminates abnormal cells; (2) Equilibrium, describing a process (that may occur over several years) during which the IS maintains a selection pressure (in an evolutionary sense) on tumor cells, which in combination with increasing genetic instability (mutations that generate cell heterogeneity), promotes the appearance and survival of tumor cell variants with augmented capabilities to avoid or suppress immune actions (detection and attack); and (3) Escape, wherein the immunologically adapted tumor gradually expands without control until it becomes clinically apparent [4].

Although conceptually simple, the different processes referred to as CI are extremely complex and dynamic, since the interaction between the IS and tumor cells involves numerous components from both the Innate and the Adaptive IS, and the behavior of these components, as well as that of the tumor cells, changes over time. From a practical point of view, the Escape phase, or rather the transition between the Equilibrium phase and the Escape phase, is of most interest because it can be considered as the point at which the IS losses effectiveness and cancer in a strict sense (a disordered replication of malignant cells in an organ tissue [5]), develops. Because of this, current immunotherapy research is focused on acquiring a better understanding of how cancer cells evade or suppress the immune response against them and on finding solutions for this tumor resistance.

### Cancer immunoediting

The interaction between Cancer Cells (CCs) and the Immune System (IS) taking place in the primary TME is quite intricate, since it involves numerous kinds of cells (see Table 1), as well as a complex exchange of chemical signals (cytokines, see Table 2) that govern their behavior [6]. Herein we provide a very brief and basic explanation of the CI stages, merely enough to serve as a technical underpinning for our model. Detailed overviews of CI can be found in [1, 2, 7]. The IS is composed of two subsystems [8]: the Innate IS (IIS) and the Adaptive IS (AIS). The IIS comprises the genetically inherited immune actions and includes cell types such as the NKs, M$\upvarphi$s and Ns that act as first responders against aberrant cells [9, 10]. The AIS evolves via a training process based on the successful immune responses to previously unknown diseases; it includes T and B cells of many varieties [11]. Other cell types that are involved in CI are DCs and, importantly, MDSCs [12, 13].

**Table 1:** Immune system elements in the TME

Cell type	Description	Role in the TME
CC	Cancer (tumor) cells	Produce antigens
NK	Natural Killer cells	Respond to tumor antigens and ﻿recruit other IS elements
M$\upvarphi$	Macrophages	Innate immune system element
M$_1$	Anti-tumor Assocd. M$\upvarphi$s	Pro-inflammatory and cytotoxic (anti-tumoral) effects
M$_2$	Pro-tumor Assocd. M$\upvarphi$s	Anti-inflammatory (pro-tumoral) and wound healing effects
N	Neutrophils	Part of the IIS; phagocytes and first responders to inflammation
N$_1$	Anti-tumor Assocd. Ns	Cytotoxic; activate T and B cells, NKs and DCs
N$_2$	Pro-tumor Assocd. Ns	Promote tumor growth, metastasis and angiogenesis
T$_{\mathrm{reg}}$	Regulatory T cells	Immunosuppessive, modulate IS ﻿and maintain tolerance
CD4+T	Helper T cells	Promote activation of other cells via cytokines and ﻿co-stimulation
CD8+T	Killer T cells	Cytotoxic; attack tumor cells, ﻿recruit other cells via cytokines
B	B lymphocytes	Present antigens and secrete cytokines
B$_{\mathrm{reg}}$	Regulatory B cells	Suppression of immune responses ﻿by production of IL-10
DC	Dendritic Cells	Present antigens to T cells and B cells
$\upgamma \updelta \,$T	Gamma delta T cells	Multiple effector (anti-tumoral) and ﻿regulatory (pro-tumoral) functions
NKT	Natural Killer T cells	Recognize CD1d molecule ﻿(an antigen-presenting molecule)
MDSC	Myeloid-derived suppressors	Interact with other IS cells to regulate their functions

Elements in the top﻿ eleven rows are included in our model

**Table 2:** Cytokines present in the TME involved in Cancer Immunoediting

Label	Description and function
IFN-$\upgamma$	Interferon produced by NKs and T cells; activates M$\upvarphi$s; ﻿induces MHC molecule expression
IFN-$\upalpha /\upbeta$	Type-I interferons, help activate and regulate the IIS; they are inhibited by IL-10
IDO	Produced in response to inflammation; immunosuppressor, limits T cells; ﻿promotes immunotolerance
Galectin-1	Regulates cell proliferation; immunosuppression by regulation of T cells; ﻿overexpression signals Escape phase
IL-6	Interleukin-6 produced by M$\upvarphi$s; inflammatory; stimulates production of N﻿s;﻿ antagonizes T$_{\mathrm{reg}}$cells
IL-10	Interleukin-10 from monocytes and T cells; enhances cytotoxin prod. by CD8+Ts; ﻿anti-inflammatory
IL-12	Interleukin-12 from DCs, M$\upvarphi$s, Ns and B cells; stimulates IFN-$\upgamma$ and TNF-$\upalpha$; ﻿anti-angiogenic; etc.
NKG2D	Transmembrane protein receptor expressed by NKs, $\upgamma \updelta \,$T and CD8+T cells; ﻿recognizes induced-self antigens
TGF-$\upbeta$	Transforming Growth Factor beta; causes immunosuppression and angiogenesis; ﻿converts T cells
TNF-$\upalpha$	Tumor Necrosis Factor released by M$\upvarphi$s to alert other cells; ﻿inflammatory
TRAIL	TNF-related ligand (cytokine) induces apoptosis in tumor cells
Perforin	Cytolytic protein found in CD4+T cells and NKs
PD-1	Programmed cell death protein 1; regulates IS, promotes immunotolerance, ﻿limits T cell inflammatory effects
CTLA-4	Cytotoxic protein; downregulates IS; expressed by activated CD4+T and T$_{\mathrm{reg}}$ cells, ﻿inhibits T cells

#### Elimination

Normal cells are transformed into CCs because of carcinogens or mutations and express tumor antigens (NKG2D ligands, MHC-I molecules, etc.) which are recognized by NKs and the cytotoxic CD8+T cells. NKs induce apoptosis in cancer cells via cytotoxic molecules (such as perforin) or antibodies, and recruit M$\upvarphi$s and Ns to dispose of dying tumor cells. DCs act as messengers between the IIS and the AIS by processing antigen material and presenting it to the elements of the AIS: CD4+T, CD8+T, NKT, and B cells. Activated T cells and NKs secrete IFN-$\upgamma$ (inflammatory) which activates M$\upvarphi$s and Ns, and inhibits angiogenesis. The activated B cells produce tumor specific antibodies while CD8+T cells induce apoptosis via cytokines and interaction with TRAIL receptors on CCs. $\upgamma \updelta \,$T, CD8+T and NK cells use NKG2D receptors to recognize induced-self antigens [6, 14]. In an immunocompetent host with a healthy IS, immunosurveillance continuously eliminates abnormal cells throughout the body and prevents the outgrowth of cancer cells. Nevertheless, some CCs can survive this immune control and enter Equilibrium.

#### Equilibrium

This is an immune-mediated state of quiescence wherein proliferation and elimination rates of CCs equal each other. This state also implies a balance between the production of anti-tumor (IL-12, IFN- $\upgamma$) and pro-tumor (TGF-$\upbeta$ [15, 16], IDO) cytokines. The IS continued response is mostly carried out by CD4+T and CD8+T cells, with participation of NKs and T$_{\mathrm{reg}}$ cells. Most importantly, during the Equilibrium phase tumor cells undergo an evolutionary process (often described as editing or sculpting) in which the IS activities constitute a form of environmental pressure and mutations act as adaptations to said environment (i.e. Darwinian selection leading to survival of the fittest). The Equilibrium phase may occur throughout several years during which poorly/non-immunogenic and immunosuppresive transformed cell variants emerge [7, 17, 18]. At this point the IS losses effectiveness and the Escape phase begins.

#### Escape

This phase is characterized by tumor growth without hindrance from the IS. For this to occur, a number of complementary and intricate mechanisms are set in motion that essentially causes the TME to function in a diametrically different manner to what it would normally do. CCs implement strategies to: (1) Evade immunorecognition by decreasing their expression of antigens and increasing that of anti-apoptotic molecules; (2) Suppress immune function by secreting cytokines such as TGF-$\upbeta$, IL-6, IL-10 and NKG2D ligands (that inhibit the cytotoxicity of $\upgamma \updelta$T cells); and (3) Recruit immune cells to indirectly promote tumor growth (also by suppressing immune function). Recruited cell types include B, B$_{\mathrm{reg}}$, NKT, NK, $\upgamma \updelta$T and MDSCs (that suppress the activation of T and NK cells) [4, 19].

### Scope and contributions

In previous paragraphs, the phases of CI have been conceptualized as dynamic scenarios wherein two rivaling forces face each other. In the Elimination phase the IS prevails over tumor cells; during the Equilibrium phase the opposing forces remain balanced; in the Escape phase tumor cells triumph over the IS. This is of particular significance for the present work because herein we propose to characterize the IS-tumor interaction by means of an energy-based model developed for the study of phase transitions. Specifically, we employ the Ising-model Hamiltonian [20] which we have used before for the analysis of complex interactions between opposing agents [21, 22] and apply it to the analysis of CI. To the best of our knowledge, this is the first model of CI that employs this energy-based approach. Note that the “energy” to which we refer is a unitless measure used to characterize computational models^1^ not to be understood as a physical quantity.

Our proposal is developed in the context of systems biology [8, 23, 24] as an Agent-Based Model (ABM) where emergent behavior is driven by Gompertzian growth and the probabilistic interactions between the agents. Just as any model describing the intricate IS-tumor interaction, wherein many processes are not completely understood yet, the present proposal introduces simplifications in order to make the model manageable and understandable: we disregard the tumor physical structure which gives place to further categorization of the CCs according to their location inside a solid tumor; also, this work is limited to the avascular phase of tumor growth. Such aspects have been discussed elsewhere [25–29]. We emphasize that the main objective of modeling and simulation of CI is to promote the scientific understanding of this complex process, by the generation of emergent behavior *in silico* that is very difficult to observe directly *in vivo* or even *in vitro*. In the following section we offer a brief recount of relevant prior efforts towards said objective.

## Related work

The tumor-immune system interaction in the TME can be formalized as a biological system [23]. Computational models of such a system are of great importance, since the analysis made possible by simulations can improve our comprehension of the process behind the disease [21, 30, 31].

In the scientific literature on cancer tumor growth [32], as well as on that related to the IS response [33, 34], the system biology modeling is carried out under either the continuous [35] or the ABM approaches [25, 36]. The continuous approaches use ordinary differential equations (ODE) [31, 37] or partial differential equations (PDE) [38, 39] to establish the parameters of tumor growth and control growth dynamics; however, frequently the required initial and border conditions are not well defined, and changes that can emerge during the process are difficult to incorporate [26]; these issues constitute main drawbacks of this approach.

In the ABM approach, simple agents represent the elements of cancer and of the IS [40]; the interaction between these results in a complex emergent behavior which is not restricted to the linear combination of the individual elements [41, 42]; hence ABMs can incorporate the changes that bring about non-deterministic interactive processes [21, 25, 36].

In 2006, Novozhilov et al. [35] described a deterministic model for oncolytic viruses used as anti-cancer therapy. The Lotka-Volterra logistic equations were employed to model the spread of the virus infection in the tumor, and the high proliferation of CCs followed an exponential growth-function at early stages of tumorigenesis. Using the model of the virus therapy it was concluded that both the infected and uninfected tumor cells could be eliminated over time, even to complete recovery.

Studies of in-vitro stimulation of T cells for patient treatment have addressed the loss of immunocompetence with age, particularly to fight cancer. Figueredo and Aickelin [34] employed an ABM simulation to show that the processes of immune system aging causes the populations of naïve T cells (those able to respond to novel pathogens) to decay over time; aging affects the naïve T cells response against cancer as well as the response to the anti-tumour vaccination process.

In 2012, Wilson and Levy [37] reported experiments that suggest that TGF-$\upbeta$ inhibition could amplify the anti-tumor immune response when combined with a tumor vaccine. The ODE-based model of cooperative interaction follows the dynamics of the tumor size, TGF-$\upbeta$ concentration, cytotoxic and T$_{\mathrm{reg}}$ cells.

In 2015 Wells et al. [43] developed a hybrid discrete-continuous computational model of a nascent metastatic tumor to investigate how functional and spatial heterogeneity of cell types impact tumor pathogenesis. They discovered that tumor escape was enhanced by heterogeneity in the responses of individual immune cells to their environment. The authors carried out simulations assuming deterministic or stochastic polarization of M$\varphi$s. Stochastic polarization was modeled by^2^: $p(M_2)=0.5(1+erf((-1+MS2/p13)/p16))$ where *erf* is the error function, *p*13 is a threshold on *MS*2 (an effector cytokine) and *p*16 is a stochasticity parameter; in turn, $p(M_1)=1-p(M_2)$. Although the model of Wells et al. is limited to very specific mechanisms (such as macrophage functional polarization occurring within 5 days of tumor implantation), their work is noteworthy because it presented multi-parametric sensitivity analyses through which the capabilities of ABMs to build an understanding of the phenomena being modeled are illustrated.

Ku-Carrillo et al. [44] described a model of cancer tumor growth that includes the IS response, which is weakened by the effects of obesity and highly-caloric diets. Their model employs ODEs and logistic growth of the amount of fat stored in the adipocyte cells of the patients.

In 2017, Pourhasanzade et al. [25] presented an ABM of tumor growth with some resemblance to this work. Their study discussed the immune response to prevent cancer development, focusing on the growth of solid tumors; in contrast, our proposal models more of the IS elements and employs an energy-function to evaluate the state of the system. Thus, these two works could be considered complementary.

In 2019 Norton et al. [6] summarized the applications of ABM and hybrid modeling to the TME and cancer immune response, with an emphasis on intra-tumor heterogeneity and interactions between cancer cells and stromal cells, (including immune cells), on the tumor-associated vasculature in relation to the immune response, and on cancer immunotherapy. In their review, the approaches to model the immune system are broadly categorized into top-down and bottom-up models; top-down approaches include those based on ODE, PDE and stochastic differential equations (SDE), and model population of cells and their mean behavior at the macroscopic level. In contrast, the bottom-up approaches track individual cells (or other microscopic elements) and their interactions, from which complex emergent behaviors arise. Although features such as stochastic behavior and heterogeneity are easier to capture via bottom-up models, these require more computational power to track the individual agents, and thus the number of agents that can be modeled is a function of the computational resources available.

Nuñez-López et al. [45] studied the interplay between tumor cells and the IS starting from a deterministic model and transforming it into an SDE model whose simulations were related to the phases of CI. Previously, other works had compared models based on SDEs, ODEs and PDEs to ABMs, notably Figueredo et al. [46, 47]. In these works, tumor proliferation and death were defined by $p(T)=aT^\alpha$ and $d(T)=bT^\beta$, respectively, where *T* represents the number of tumor cells. The transition rates for proliferation and death are given by the logistic growth $aT(1-Tb)$ and the authors described the parameters and behaviors of agents involved in tumor growth as follows:

**Table:** 

Parameters	Reactive behavior	Proactive behavior
*a*, $\alpha$, *b* and $\beta$	Dies if *rate* $< 0$	Proliferates if *rate* $> 0$

where *a*, *b*, $\alpha$ and $\beta$ are parameters that can vary substantially, depending on the case study. A tumor cell agent thus possessed two possible states, *alive* or *dead*, and would move between states through two possible actions, *dies* or *proliferates* (clearly, if an agent moves to state *dead* it will remain in that state). As can be seen, one advantage of ABMs is the simplicity of the agents and of the imbedded rules for individual behavior.

Very recently, Sajid et al. [48] studied cancer niche construction by means of a 2-D Ising model to capture the interaction between clusters of cancer cells and of healthy cells, employing the Kikuchi free energy approximation to describe the spatiotemporal evolution of said model. Without pointing out a direct connection, their study describes the building blocks required for CI: apoptosis (or cell death, required in Elimination), local cancer growth (a state akin to Equilibrium since cancer is kept local), and metastasis (which occurs in the Escape phase). However, Sajid et al. do not discuss CI itself, their model is not an ABM and they employ a different formulation of the system Hamiltonian, more adequate for their purposes, instead of the mean field approximation [21] used in our study.

The computational model presented herein is a hybrid proposal between ABM and the continuous approach, since the behavior of the different agents in the simulated system is governed by relationships derived in both the continuous and the discrete domains, including a logistic growth function (a main choice in the ODE- or PDE-based modeling) for the cancer and IS elements, and the Ising-model Hamiltonian [20] to characterize the interaction of agents as cooperation among partners or confrontation between opponents [21, 22]. We conclude by pointing out that the works of Torquato [49], Barradas et al. [21], Sajid et al. [48], and the present study can be seen as complementary to each other, and together describe the basis for an energy-based computational model of cancer from the immunological perspective.

## Agent-based model of cancer immunoediting

In this work, an Ising model energy function is employed to characterize the interaction between cancer-cells and IS-cells within an agent-based framework. A good model should allow us to generate and observe the different phases of Cancer Immunoediting, while avoiding an inordinate amount of control parameters. Our model requires only the setting of four hyperparameters; after that, the model dynamics and the end result depend entirely on the stochastic behavior occurring in the simulator according to the three main components described below.

### Modeling cancer immunoediting

**Fig. 1:**
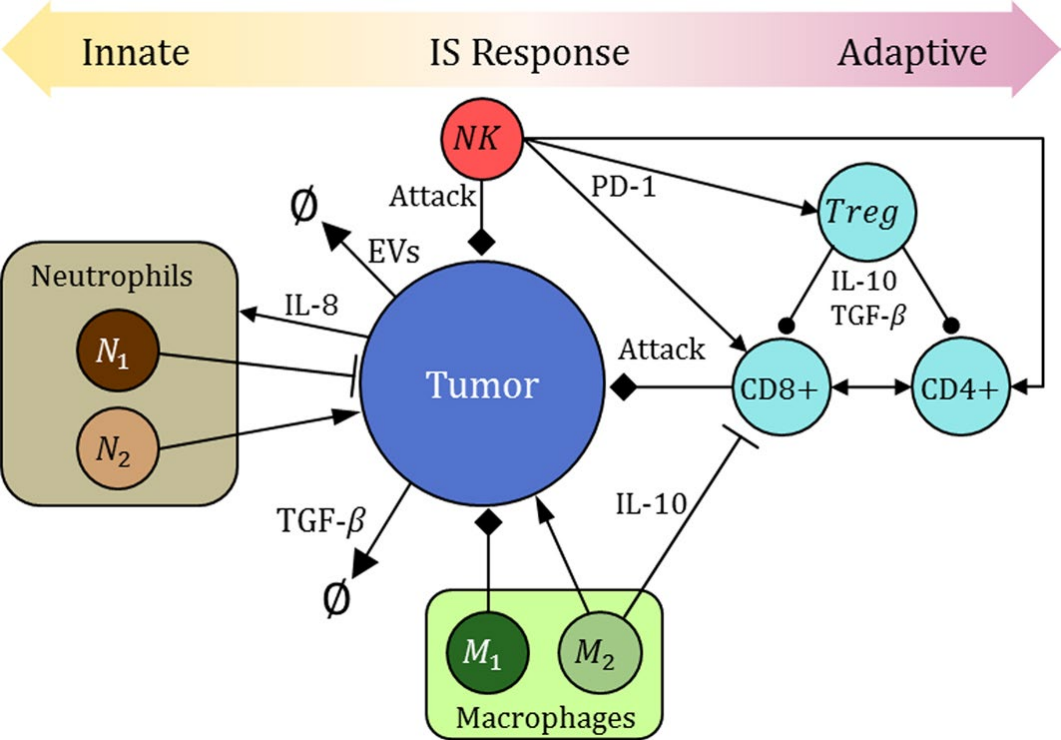
IS-Cancer interaction in TME: some Ns and M$\upvarphi$s can become pro-tumoral. T$_{reg}$ cells regulate CD8+T and CD4+T cells. NKs attack CCs and promote T cells

We study a simplified version of the cancer versus IS interaction in the TME, for which the biological system modeled is depicted in Fig. 1 and the corresponding elements are listed in Table 1. In our ABM there are agents representing these elements, with associated rules that govern their behavior. The simulation of the system is carried out by sequentially and iteratively invoking the different types of agents, according to the pseudocode in Algorithm 1. The actions of agents are probabilistically defined, and the complex behavior of the system which we want to characterize arises from their interaction with other agents of different types. Said interaction becomes intricate by the emergence of pro-tumor actions from IS elements. For instance, added complexity occurs when M$\varphi$s and Ns are “recruited” to inhibit immune functions (by release of inhibitory cytokines), becoming pro-tumoral [11].

Although it has been simplified, our model contains numerous parameters, the most important of which are listed in Table 3. Since adjusting each of these parameters individually is undesirable, our approach is to define a small set of four *hyperparameters*, based on which the values of all the parameters can be set automatically. Internally, all of the parameters are given individual values, but to the user this process is transparent. A detailed explanation of parameter initialization is provided in the “Simulation methodology” section.

**Table 3:** Model parameters of interaction

ID	*Cancer growth parameters*	Notation
1	No. of initial tumor cells	$C_n$
2	Transforming Growth Factor TGF—$\beta$	$\beta$
3	Pr. of antigen production by CCs	$p_1$
*Innate IS parameters*		
4	No. of initial NKs	K
5	No. of initial M$\upvarphi$s	M$_1$
6	No. of initial Neutrophils	N$_1$
7	Pr. of recruiting NKs	$p_{2}$
8	Pr. of recruiting M$\upvarphi$s	$p_6$
9	Pr. of recruiting Neutrophils	$p_{1,3}$
10	Pr. of NKs attacking CCs	$p_{A8}$
11	Pr. of M$_1$ cells attacking CCs	$p_{A6}$
12	Pr. of N$_1$ cells attacking CCs	$p_{A3}$
13	Pr. of M$\upvarphi$s becoming M$_2$	$p_{A7}$
14	Pr. of Neutrophils becoming N$_2$	$p_{1,4}$
15	Pr. of M$_2$ cells promoting tumor cells	$1 - p_{A6}$
16	Pr. of N$_2$ cells promoting tumor cells	$1 - p_{A3}$
17–21	Max. age of M$_1$, M$_2$, N$_1$, N$_2$, and NK cells	$A {_{\mathrm{M}_1}}, A {_{\mathrm{M}_2}}, A {_{\mathrm{N}_1}}, A {_{\mathrm{N}_2}}, A {_{\mathrm{K}}}$
*Adaptive IS parameters*		
22	No. of initial CD8+T cells	T
23	No. of initial CD4+T cells	T$_\mathrm{_h}$
24	No. of initial T$_{\mathrm{reg}}$ cells	T$_{\mathrm{reg}}$
25	Pr. of recruiting CD4+T cells	$p_{8}$
26	Pr. of recruiting CD8+T cells	$p_{7}$
27	Pr. of recruiting T$_{\mathrm{reg}}$ cells	$p_{9}$
28	Pr. of T$_{\mathrm{reg}}$ strengthening T cells	$u_{1}$
29	Pr. of T$_{\mathrm{reg}}$ strengthening T$_\mathrm{h}$ cells	$u_{2}$
30	Pr. of T$_\mathrm{h}$ strengthening T cells	$u_{4}$
31	Pr. of CD8+T cells attacking CCs	$u_{3}$
32–34	Max. age of CD8+T, CD4+T, and T$_{\mathrm{reg}}$ cells	$A_\mathrm{T}, A{_{\mathrm{T}_h}}, A{_{\mathrm{T}_{reg}}}$

“Pr.” stands for “Probability”

**Fig. 2:**
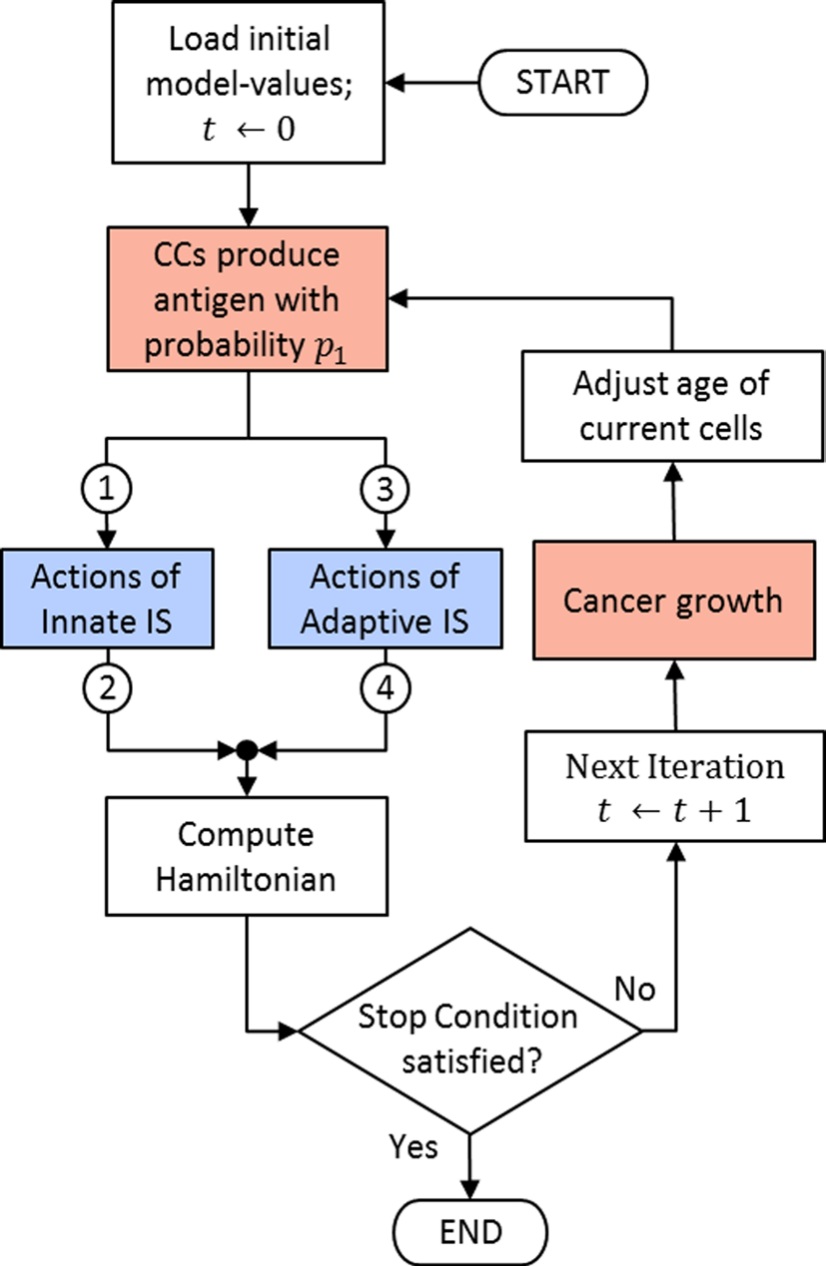
System flowchart. Points numbered 1 to 4 indicate entry and exit points to the diagrams in Figs. 3 and 4. Blue and red indicate mostly anti-tumoral and pro-tumoral processes, respectively

**Fig. 3:**
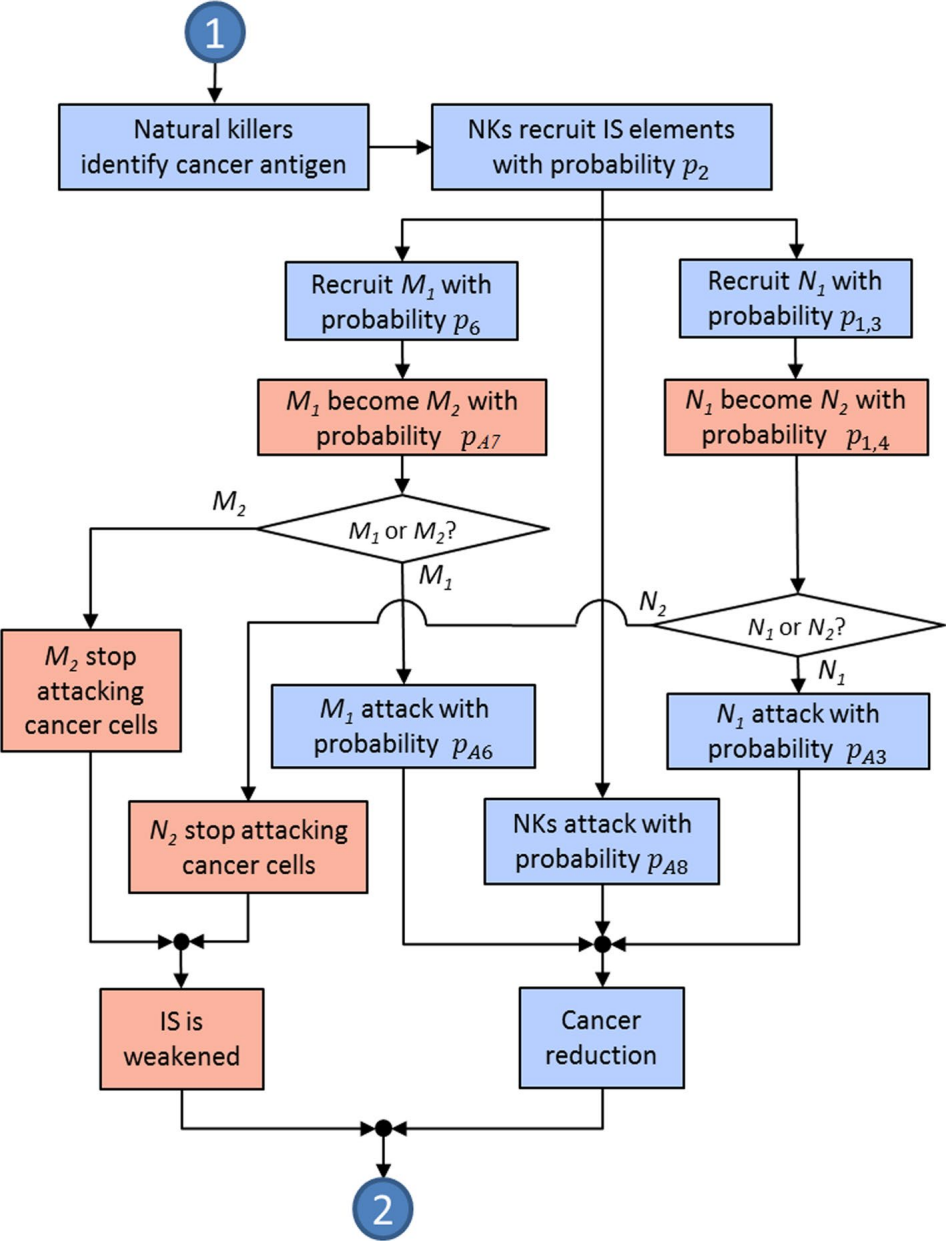
The cancer growth and the IIS response at the micro-environment

**Fig. 4:**
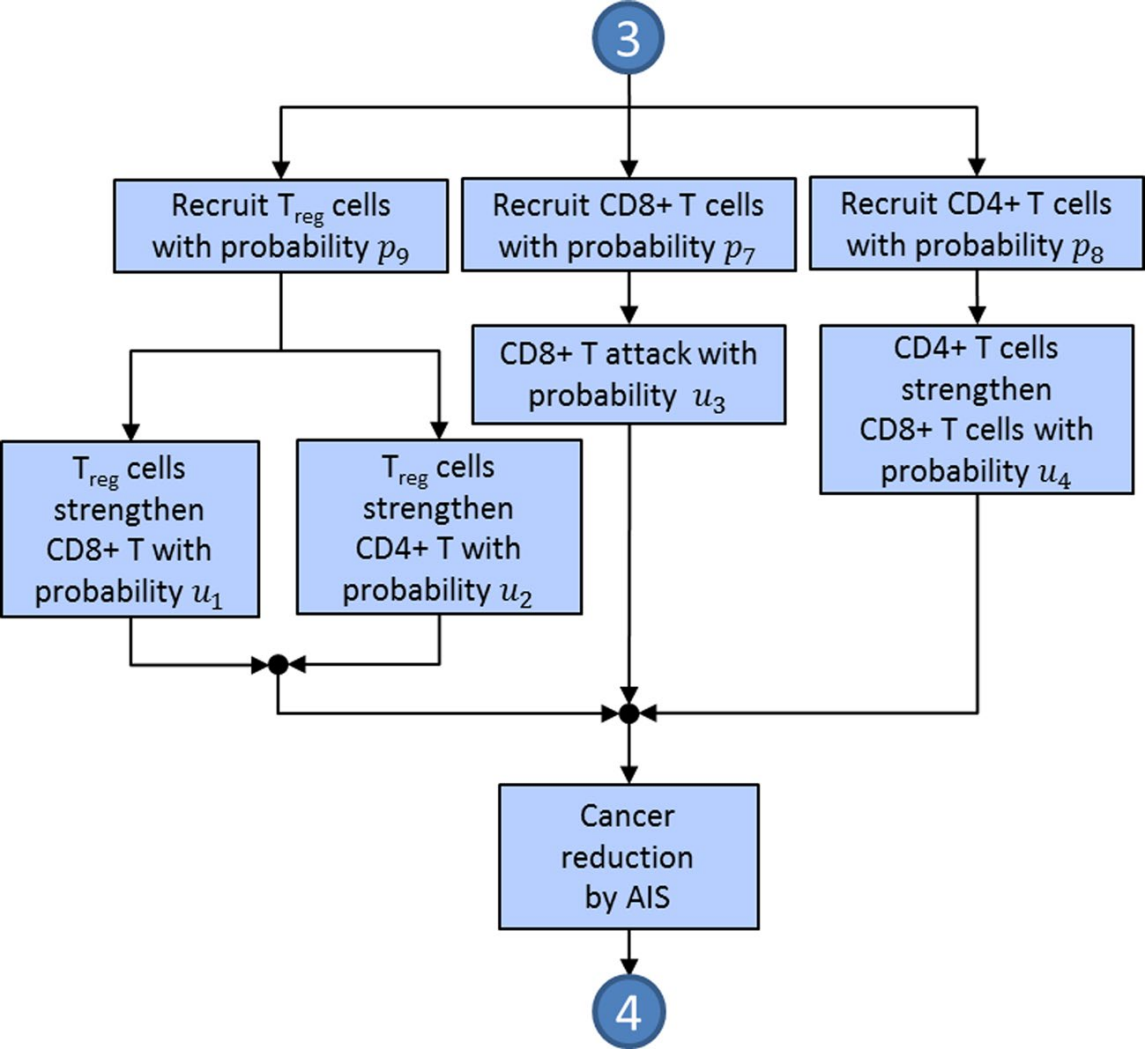
The tumor immune interaction in the TME with the AIS elements CD8+T, CD4+T and T$_{\mathrm{reg}}$ cells

The probabilities referred to in this subsection regulate the behavior of the elements involved in the TME. The actions to be carried out by the cancer and IS elements are summarized in three diagrams. A complete system flowchart is shown in Fig. 2; the cancer-IIS interaction is summarized in Fig. 3, and the cancer-AIS interaction is described in Fig. 4.

For every possible action to occur, certain conditions must be met, which are defined in terms of the corresponding probabilities. Over time, and as a result of the different actions, the number of CCs and IS cells will change, and with them, the energy of the system, characterized by the Ising-like Hamiltonian (see “Characterizing the stochastic interaction” section).

If the CCs are not eliminated by the anti-tumor elements of the IS, the rate of tumor growth will depend on the strength of the tumor (expressed through the TGF-$\upbeta$) and on the Ns and M$\upvarphi$s that become pro-tumoral. Meanwhile, the strength of the IS response depends on the age and health of its elements (NKs, Ns and M$\upvarphi$s) and those of the AIS (CD4+T, CD8+T, and T$_{reg}$ cells). If not killed by other agents, the active period of the cells will be probabilistically adjusted based on their age.

It has been suggested that the probability of successful interaction between CCs and the anti-tumor neutrophils N$_1$ is complementary to the probability of interaction between tumor cells and the pro-tumor neutrophils N$_2$ [11]. A similar relationship holds for the probability of interaction between the tumor cells and the anti-tumor macrophages M$_1$ and the complementary probability of interaction between the tumor cells and the pro-tumor macrophages M$_2$.

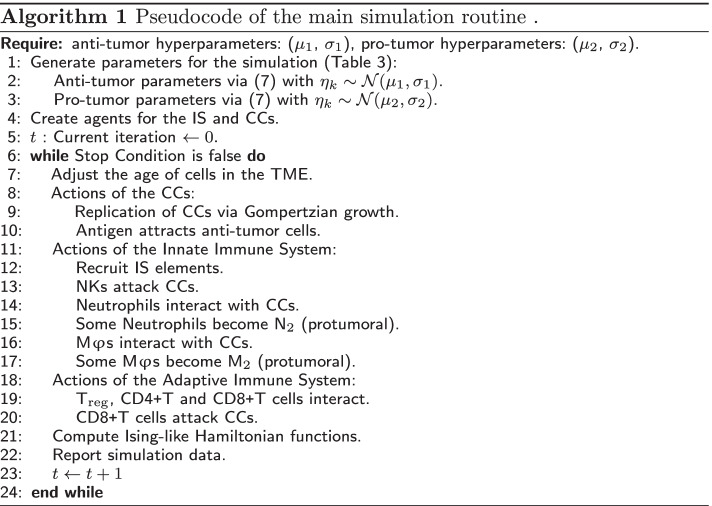



### Modeling growth in cancer immunoediting

**Fig. 5:**
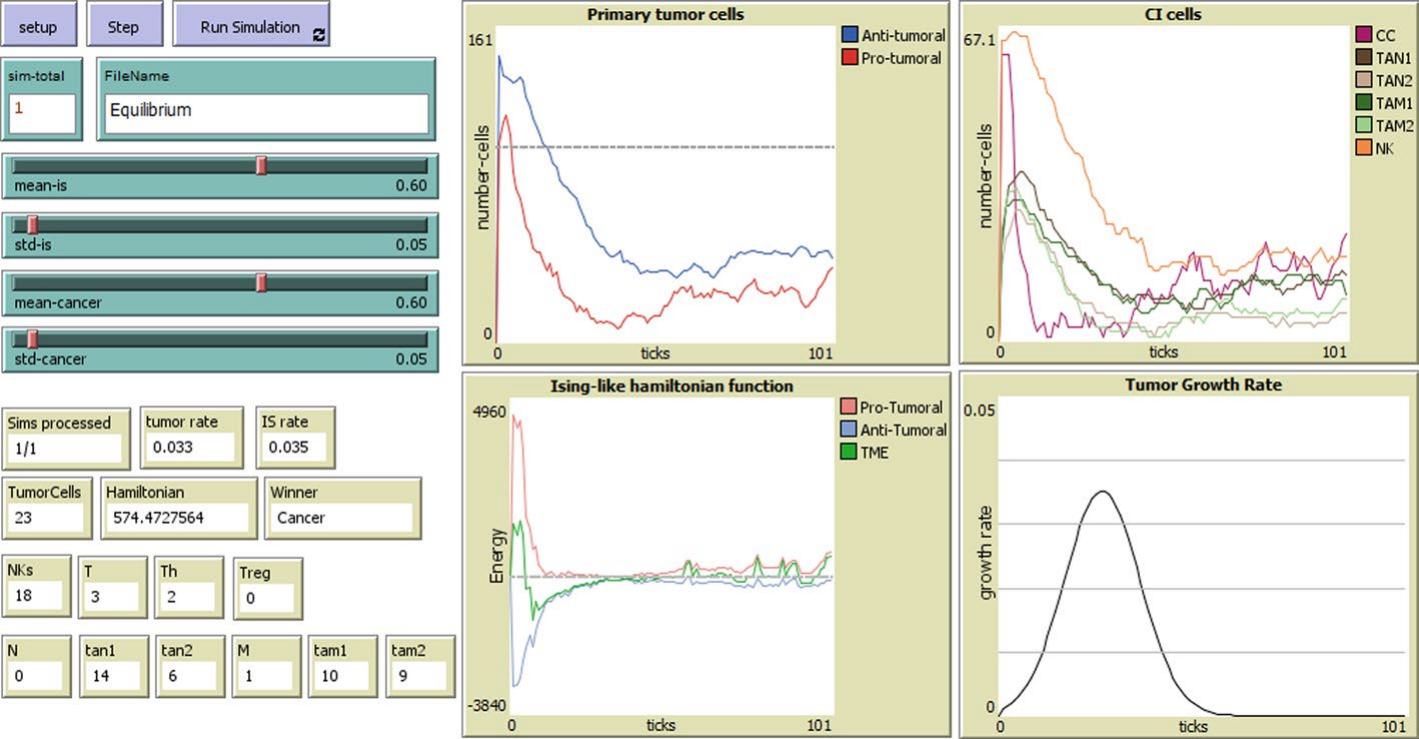
Interface of our simulator with results of one simulation illustrating the Equilibrium phase

A needed complementary component in the above description is a model for the tumor growth and for the recruitment of IS elements, which define the strength of the cancer and of the immune response, respectively. We describe the growth of the cell populations by means of logistic functions, which approximate Gompertzian growth, a popular choice in modeling growth patterns, in particular for modeling the population of cells in tumor growth [39, 50–52]. The logistic function *f*(*t*) with midpoint $t_0$, maximum value of *a*, and growth rate *k*, is given by:

1 $$\begin{aligned} f(t) = \frac{a}{1 + e^{-k(t - t_0)}} \end{aligned}$$

which has $\mathbb {R}$ as the usual domain, but as time is positive, we translate it to $\mathbb {R}^{0+}$ (see the bottom right plot in Fig. 5).

Having defined the growth of a cluster of cells as *f*(*t*), the growth rate is given by the derivative of said function; letting $\varphi (t)=1/(1+e^{-k(t-t_0)})$ we have:

2 $$\begin{aligned} f'(t) = a\varphi '(t)=\frac{-a\left( 1+e^{-k(t-t_0)} \right) '}{\left( 1+e^{-k(t-t_0)} \right) ^2}= \frac{ak \left( e^{-k(t-t_0)}\right) }{\left( 1+e^{-k(t-t_0)}\right) ^2} = ak\varphi (t)(1-\varphi (t)) \end{aligned}$$

Through $\gamma (t):= f'(t)$ (a logistic distribution), the size of the cell clusters can be updated over time by means of the expression:

3 $$\begin{aligned} n(t + \Delta t) = \gamma (t + \Delta t)n(t) \end{aligned}$$

where $\Delta t$ represents a positive interval of time of arbitrary length, and the domain of *n*(*t*) is also $\mathbb {R}^{0+}$. The cell clusters are revisited in (5) and (6) below.

### Modeling actions and interactions of agents

To model the actions of agents listed in Algorithm 1, first let us distinguish between actions involving two agents, called interactions, and actions involving only one agent, like cell aging or one anti-tumoral cell becoming pro-tumoral. For all interactions, an agent is invoked and paired with a probabilistically-chosen second agent of adequate target-type (for instance, a M$\varphi$ is paired with a CC) and their interaction (with anti- or pro- tumoral effect depending on the polarization of the M$\varphi$ in this example) occurs according to the following rule:

4 $$\begin{aligned} \text{Interaction} = {\left\{ \begin{array}{ll} True, &{} p_k \ge r, r \in [0, 1]\,\text{is a uniform random variable.} \\ False, &{} \text{otherwise.} \end{array}\right. } \end{aligned}$$

where $p_k$ represents the applicable probability from those listed in Table 3. Equation (4) describes a generic rule for interactions. The particular interaction rules are described in Table 4, among the rules for single-agent actions.

**Table 4:** Rules for actions and interactions between agents (${\mathcal {N}}$: Normal distribution)

*Cancer growth*	
Cancer growth through (1) with $a=10\,\beta -1, \,k=4, \,t_0 = 0.5,\, \beta \sim {\mathcal {N}}(\mu _2,\sigma _2)$	

Innate IS response with $\eta _k\sim {\mathcal {N}}(\mu _1,\sigma _1)$	
Generate Innate IS cells: $\{\text{K}, \text{M}_1, \text{N}_1\}= 100\times \{\eta _4:\eta _6\}$	
Recruiting of IS cells through (4) with $\{p_2,p_6,p_{1,3}\}=\{\eta _7,\eta _8,\eta _9\}$	
Success of attack by IS cells through (4) with $\{p_{A_8}, p_{A_6}, p_{A_3} \} = \{\eta _{10}, \eta _{11}, \eta _{12}\}$	
M$\upvarphi$s and Ns become pro-tumoral with $\{p_7, p_{1,4}\} = \{\eta _{13},\eta _{14}\}\sim {\mathcal {N}}( \mu _1 / (\mu _1 + \mu _2), \sigma _1)$	

Adaptive IS response with $\eta _k\sim {\mathcal {N}}(\mu _1,\sigma _1)$	
Generate Adaptive IS cells: $\{\texttt{CD4+}T, \texttt{CD8+}T, \text{T}_{\mathrm{reg}}\}= 100\times \{\eta _{22}, \eta _{23}, \eta _{24}\}$	
Recruiting of T cells through (4) with $\{p_7,p_8,p_9\}=\{\eta _{25},\eta _{26},\eta _{27}\}$	
T-cells strengthen each other using (4) with $\{u_1,u_2,u_4\}=\{\eta _{28},\eta _{29},\eta _{30}\}$	
Success of CD8+T cells attacking CCs (4) with probability $u_{3} = \eta _{31}$	

Aging of the IS cells leads to loss of efficiency against cancer [34]. In this work, individual cell age is modeled by dividing a cell’s life into three parts: the first third includes the time from cell division to the beginning of maturity; the second third comprises the period of time during which the cell is fully developed and works most efficiently; the last third includes the cell’s aging and death; T$_{reg}$ cells have a role in regulating or suppressing other cells in the IS, such as CD4+T and CD8+T cells [53]. Below we describe the way in which these interactions are characterized.

### Characterizing the stochastic interaction

The Ising model is a classic in the formalization of ferromagnetism that arises from the interaction between molecule spins [14]; it is a simple model from statistical physics used to describe energy interactions and phase transitions of matter. The 2-D Ising model Hamiltonian *H*, given in (5), is increasingly employed in the modeling of complex processes in biology, chemistry and their interdisciplinary matters [49, 54]. In our present context, $x_i$ and $x_j$ represent two interacting clusters of cells, belonging to the tumor and to the IS. The Hamiltonian quantifies the energy involved in the interaction between those clusters, which determines the outcome of the interaction, i.e. whether the CCs or the IS cells dominate the interaction.

5 $$\begin{aligned} H = -\frac{1}{2}\sum _{i,j}^{n}w_{ij}x_{i}x_{j} - \nu \sum _{i}^{n}h_{i}x_{i} \end{aligned}$$

The characterization of each cluster of cells is given by:

6 $$\begin{aligned} x_i = c_i n_i \end{aligned}$$

where $c_i \in \{1, -1\}$ according to whether the cluster $x_i$ acts in favor of the IS or the cancer, respectively. The variable $n_i$, computed by (3) is the number of cells in $x_i$; notice that this may refer to groups of cells or to a whole population of them.

The energy in (5) is also determined by variables $w_{ij}$, which weight the interaction of cells that cooperate or antagonize each other as members of $x_i$ or $x_j$. Finally, $\nu$ denotes the whole (magnetic in ferromagnetism) field energy, and $h_i$ the way in which this affects each cluster of cells $x_i$. In the present application, these parameters are related to the effect of the combined chemical signals in the micro-environment. The specific values for the weights and the field energy require to be specifically tuned to experimental data, which for simplicity is not used in this work. Instead we set $w_{ij}=1$ and $\nu =0$, which will allow us to analyze the unweighted and unbiased interaction of the elements in our system.

The model Hamiltonian (5) is a suitable measure of the model energy, which indicates the dominating side in a system of opposing forces at any time during the simulation.

## Simulation methodology

With the purpose of demonstrating the potential uses of our proposed model, we carried out simulations to illustrate how the model can be employed to analyze the interaction between cancer cells and the immune system through time. In particular, we are interested in showing that the model is capable of generating the different phases of Cancer Immunoediting, that the phases occur for reasonable values of the model’s hyperparameters, and that these phases can be correctly characterized by the model’s Hamiltonian. The model was implemented in the NetLogo multi-agent modeling environment [55] and includes a graphical interface through which a user can input the model’s hyperparameters and observe the outcome of one individual simulation as a set of plots (Fig. 5) that show (a) the number of anti-tumoral and pro-tumoral cells through time; (b) the number of each type of cell in the CI system; (c) the Hamiltonians for the anti-tumoral and pro-tumoral portions as well as the total Hamiltonian in the TME; (d) the tumor growth rate.

The four hyperparameters, two for the IS $(\mu _1, \sigma _1)$ and two for the CCs $(\mu _2, \sigma _2)$ can be introduced into the simulator by means of slider controls; these variables can be set to any values between 0 and 1 to define two Normal probability distributions. Next, scaling factors $\eta _k\in [0, 1]$ are sampled from the distributions defined by $(\mu _1, \sigma _1)$ or by $(\mu _2, \sigma _2)$ depending on whether the *k*-th parameter (of those listed in Table 3) relates to the IS cells or the tumor cells, respectively. In this way, the individual probabilities and strength levels of the elements of the IS and the cancer can be adjusted automatically, by setting:

7 $$\begin{aligned} \text{Parameter}_k = \eta _k\cdot b_k \end{aligned}$$

where $b_k$ is the base value for $\text{Parameter}_k$.

Notice that this procedure is not part of the model, but a methodology to simulate the model under different sets of parameters to carry out the system characterization. A similar procedure was followed in [43] to characterize the TME network robustness and the role of heterogeneities, but it was done by sampling the values of their parameters through a linear distribution. In this study it is more convenient to sample the parameter values under Normal distributions centered around reference values chosen for each of the phases of CI, because this facilitates the organization of the simulations and allows us to control the variance of initial parameters between simulations; specifically, for all the simulations we fixed $\sigma _1= \sigma _2=0.05$. Keeping the variance of the initial parameters fixed becomes relevant for the analysis of the results, because then the observed differences in variability between CI phases can be attributed to the functioning of the model, rather than to the way in which the initial parameters were distributed.

**Fig. 6:**
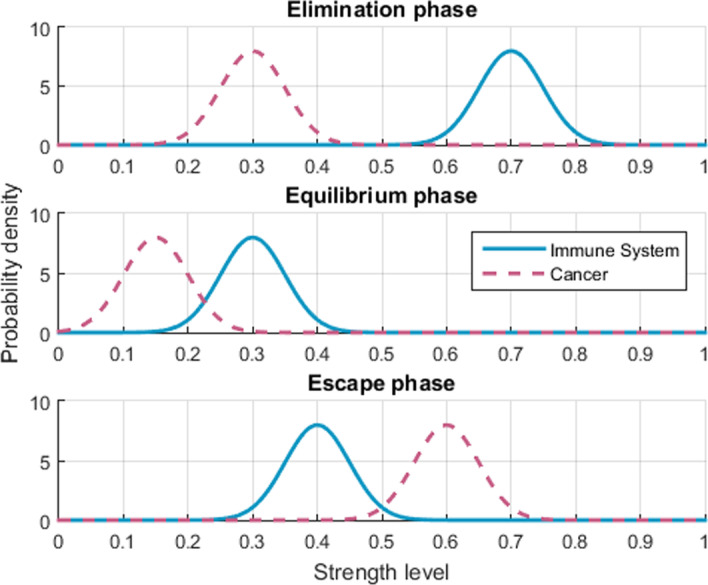
Hyperparameter distributions used to model the three phases of CI

As stated, herein we are particularly interested in generating the scenarios corresponding to the three phases of Cancer Immunoediting: Elimination, Equilibrium and Escape. For the Elimination phase the strength of the IS was set significantly higher than that of the cancer ($\mu _1 = 0.7$, $\mu _2 = 0.3$) since the objective is to simulate an IS operating optimally by consistently eliminating a moderate amount of abnormal cells. For the Equilibrium phase the strength of both subsystems was lowered and set much close to each other, with that of the IS still being higher than that of cancer ($\mu _1 = 0.3$, $\mu _2 = 0.15$); this corresponds to an IS that keeps in check, but just barely, a small number of cancer cells. For the Escape phase the strength of the cancer was increased, but most importantly, the strength of the IS was set to a value for which the IS remains operational although it cannot prevent the survival and reproduction of CCs ($\mu _1 = 0.4$, $\mu _2 = 0.6$). The distributions are shown in Fig. 6.

The simulations described above are designed to show the robustness of our model to variability of its parameters around central values (this variability is called Variance). In a second batch of simulations the central values (the means of the distributions used to sample the hyperparameters) were displaced to show that our model is also robust to changes of the central values (this is described as Bias). Overall, our model was tested on 15 sets of hyperparameters, and for each set of hyperparameters 50 simulations were performed to provide statistical support.

## Results

**Fig. 7:**
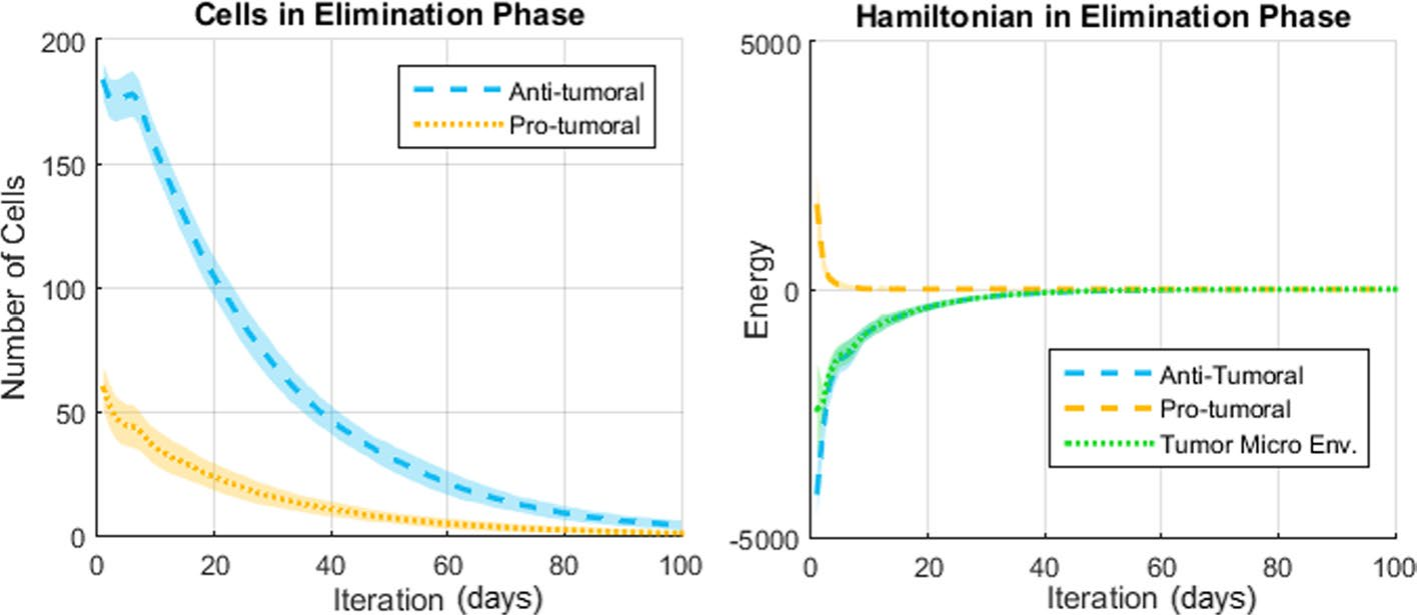
Simulation of the elimination phase of cancer immunoediting

**Fig. 8:**
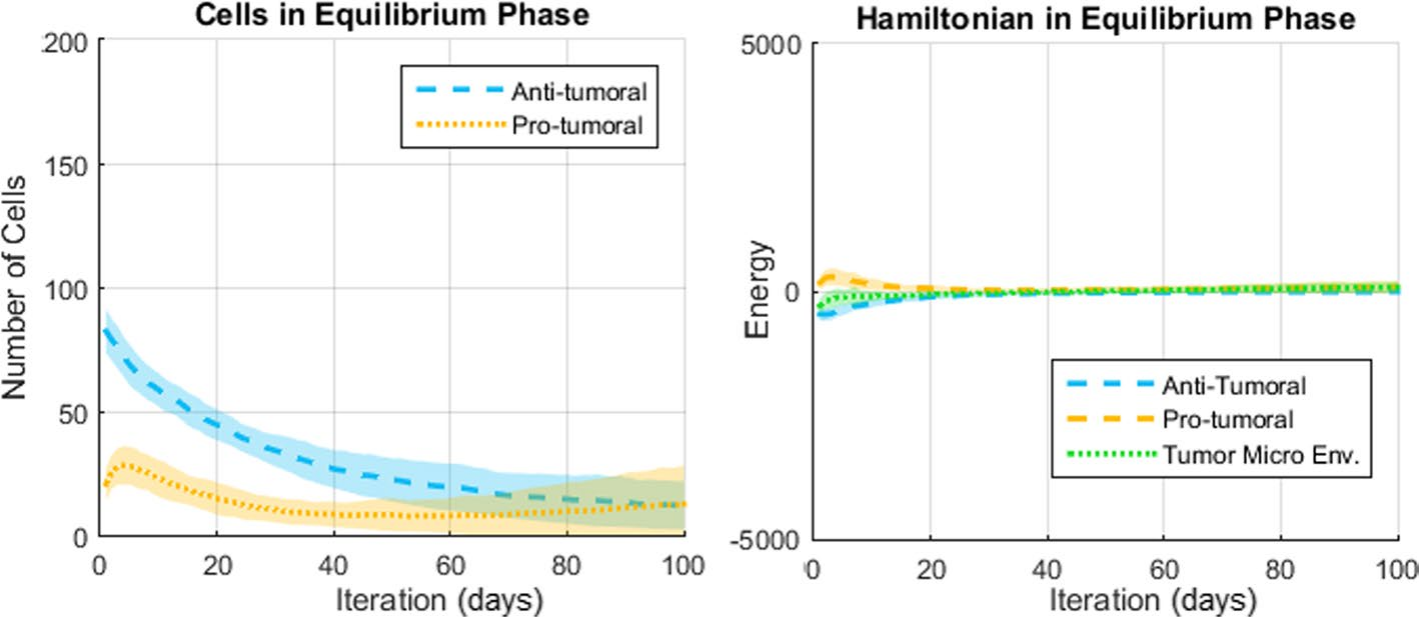
Simulation of the equilibrium phase of cancer immunoediting

**Fig. 9:**
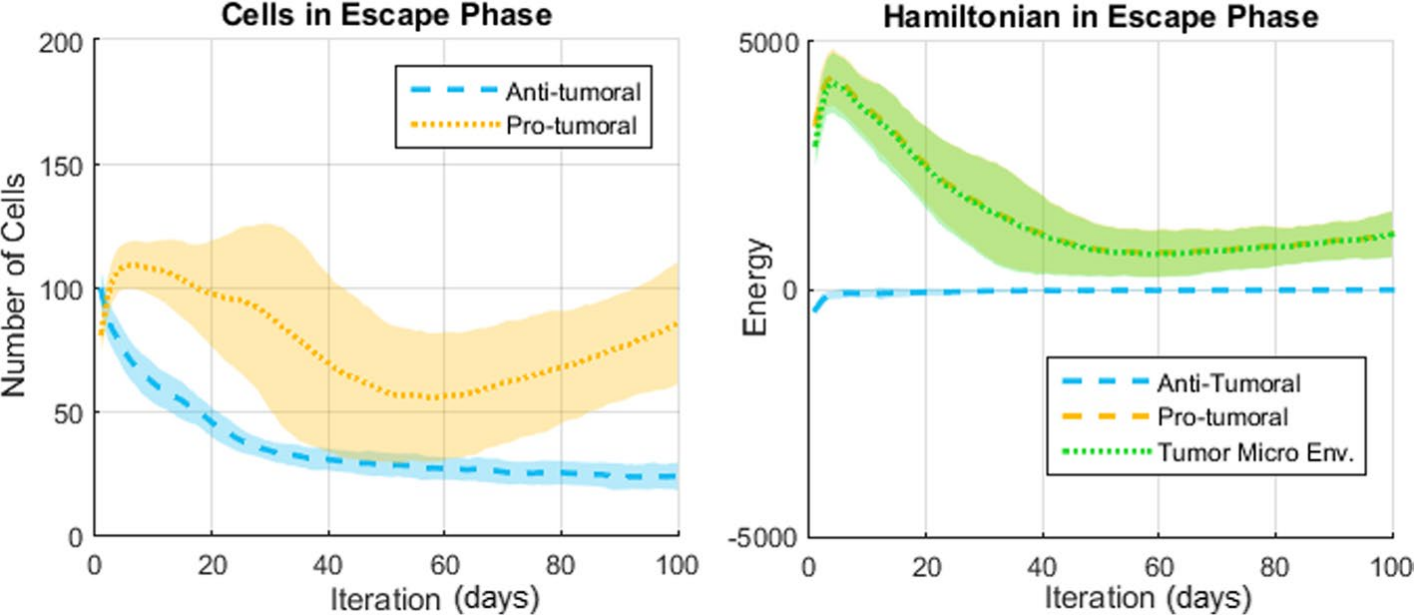
Simulation of the escape phase of cancer immunoediting

The results presented in this section come from a probabilistic system and thus a meaningful analysis must consider the average result and dispersion over many simulations. Representative results (over 50 simulations per scenario) regarding the number of cells and the Hamiltonians are shown in Figs. 7, 8 and 9 for the Elimination phase, Equilibrium phase and Escape phase, respectively. The shaded region around each curve illustrates the standard deviation of the results. The sign of the Hamiltonian is positive for the pro-tumoral agents in the system and negative for the anti-tumoral agents (signs are arbitrarily assigned and can be reversed with no effect on the final conclusion). The figures also show the sum of the two Hamiltonians: values close to zero indicate that the forces of the adversaries are nearly balanced at that particular time.

**Fig. 10:**
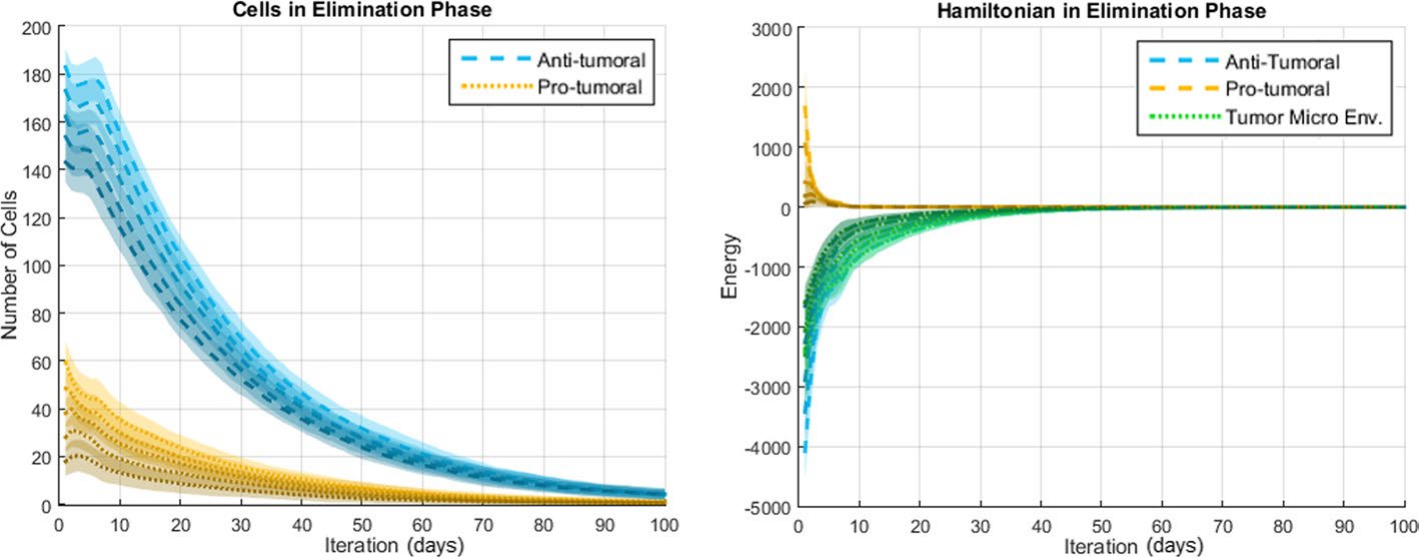
Effect of hyperparameters variations on simulation results for the elimination phase ($\mu _1 = \mu _2 + 0.40$)

**Fig. 11:**
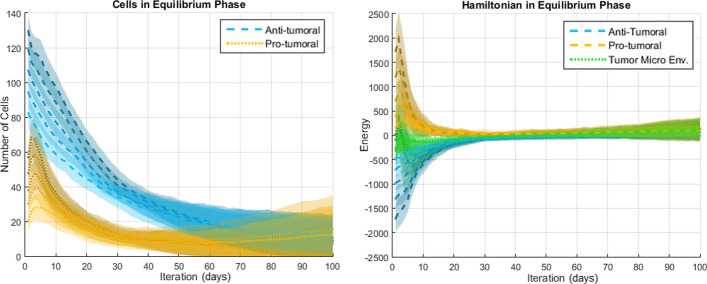
Effect of hyperparameters variations on simulation results for the equilibrium phase ($\mu _1 = \mu _2 + 0.15$)

**Fig. 12:**
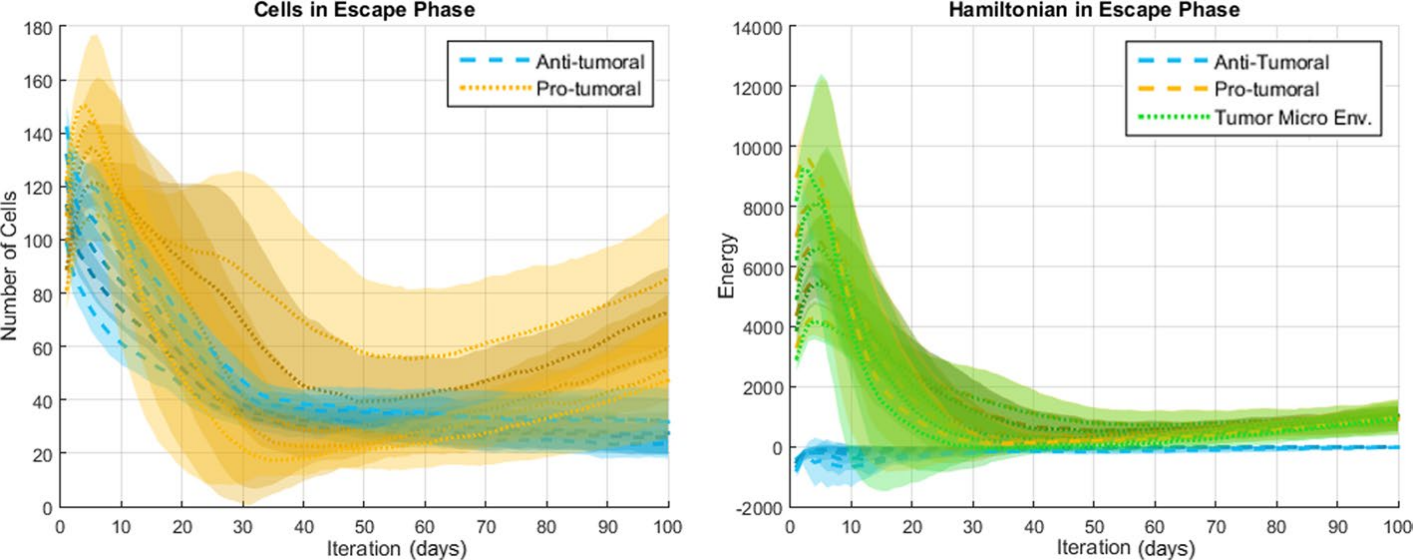
Effect of hyperparameters variations on simulation results for the escape phase ($\mu _1 = \mu _2 - 0.20$)

Figures 10, 11 and 12 illustrate the effect of using different hyperparameters on the number of cells and the Hamiltonians while simulating the Elimination, Equilibrium, and Escape phases, respectively. The objective is to observe how sensitive is our model to the setting of its control parameters.

**Fig. 13:**
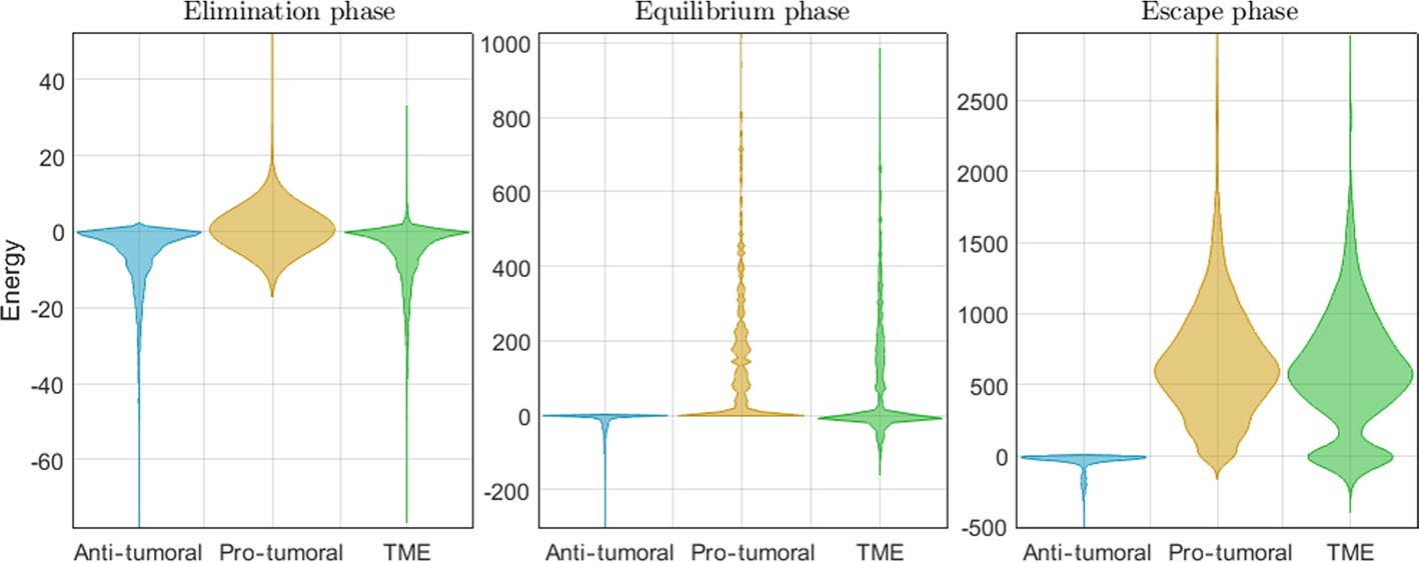
Energy probability density estimates in different phase of CI

Finally, Fig. 13 shows the probability density estimates of the energies in the system corresponding to the second half of the simulations (once the system has converged), presented as violin plots for each of the three CI phases. These density estimates were obtained via kernel density estimation [56]. Table 5 reports the means and medians of the probability densities.

**Table 5:** Central tendency statistics of probability densities: the difference between the means and the medians make the distributions in Fig. 13 left- or right-skewed

CI phase	Anti-tumoral	Pro-tumoral	TME
*Mean value*			
Elimination	− 6.5121	0.6923	− 5.8198
Equilibrium	− 19.1849	68.3668	49.1818
Escape	− 48.4486	654.6104	606.1618
*Median value*			
Elimination	− 3.1006	0	− 2.6661
Equilibrium	− 4.0000	0.6990	0
Escape	− 23.8471	616.3473	589.6464

## Discussion

Based on the results shown in Figs. 7, 8 and 9 the following observations can be made. First, with respect to the number of anti-tumoral and pro-tumoral cells, it should be noticed that the simulations start with an initial population of these cells and it is only during the first fourth of the simulations that the growth rate of CCs increases; after that, following Gompertzian growth, it decreases gradually. Thus, as time passes, the number of cells naturally tends to diminish due to apoptosis and, in the case of CCs due to elimination by cytotoxic cells (except in Escape).

During the Elimination phase (Fig. 7), an anti-tumoral strength of 0.7 versus a pro-tumoral strength of 0.3 translates into initial cell populations of about 180 anti-tumor cells versus 60 pro-tumor cells. Cell population growth is not observable, because the significantly larger number of anti-tumor cells prevents the proliferation of CCs and in turn the decreasing number of these impedes recruitment of more anti-tumor cells. Notice how the corresponding Hamiltonians reflect this behavior: the energy of the pro-tumoral agents is initially about one third of the anti-tumoral energy, then the pro-tumoral energy falls rapidly during the first few iterations and remains at a very low level for the rest of the simulation time (CCs and pro-tumoral cells have been mostly eliminated). This causes the total Hamiltonian of the TME (shown in green) to be aligned with the anti-tumoral Hamiltonian. In other words, a TME-Hamiltonian exhibiting a negative sign during the simulation shows that the system is in the Elimination phase, while the magnitude of the Hamiltonian indicates the energy of the agents’ interactions.

In the Equilibrium phase (Fig. 8), relative strengths of 0.3 versus 0.1 produce initially about 80 anti-tumoral cells and 60 pro-tumoral cells. In this case, a moderate increase in the population of pro-tumoral cells can be observed during the beginning of the simulations. As time passes this growth is counteracted by the anti-tumoral agents and the population of pro-tumoral cells remains quite stable for the remaining time of the simulations. Interestingly, as the number of anti-tumoral cells decreases substantially towards the end of the simulations, the number of pro-tumoral cells begins to show a moderate increase and the dispersion of the data grows larger. We believe that this behavior corresponds well with what is expected during the Equilibrium phase: in general and on average, the CCs are kept in check, but after balance has been maintained for a while the confrontation must be resolved either by the elimination of the CCs, or by their proliferation (leading to the Escape phase). Since the model contains stochastic elements, the resolution sometimes favors one side and sometimes favors the opposite, which is shown by the dispersion of the data. Values around zero of the corresponding TME-Hamiltonian reflect the balance between the opposing strengths from the beginning to the end of the simulations, even when the energy of the anti-tumoral and the pro-tumoral agents is not zero at the starting point.

In the Escape phase (Fig. 9), strength levels of 0.4 versus 0.6 generate initial populations of approximately 80 anti-tumoral cells and 100 pro-tumoral cells, respectively. In this case the pro-tumoral cells rapidly increase their numbers and surpass those of the anti-tumoral cells, although only up to a certain point (reaching about 110 cells on average). Then the population of pro-tumoral cells declines at varying rates up to the middle point of the simulations and grows again at a steady pace for the remaining time. Quite interestingly, the dispersion of the pro-tumoral data is significantly larger through the Escape phase than through the other two phases of CI. This is not caused by a larger spread in the sampling of the initial parameters (since the spread hyperparameter is the same as for the other phases), but is a result of the stochastic interaction in the presence of a larger population of pro-tumoral cells. The data of the TME-Hamiltonian also exhibits a larger dispersion, but a clear indication that the pro-tumor agents dominate the interaction in the TME is the fact that the Hamiltonian always shows a positive sign. In other words, a TME-Hamiltonian with a positive sign throughout the simulation indicates that the system is in the Escape phase. Also, in this phase the TME-Hamiltonian is always aligned with the pro-tumoral Hamiltonian and the magnitude of the energy is similar to that in the Elimination phase, but of opposite sign.

The results in Figs. 10, 11 and 12 show that the characteristic system behavior discussed above and corresponding to each of the CI phases can be obtained for different sets of hyperparameters: in these experiments the results were obtained using mean values for the anti-tumoral and the pro-tumoral agents with a constant difference between them (according to the equation reported in the caption of each of the referred figures). In other words, the hyperparameters’ values for which our model generates a certain phase of CI are neither unique nor arbitrary, but correspond to relationships between the values chosen for the anti-tumoral and the pro-tumoral agents that can be logically inferred from the desired system behavior. These results also demonstrate that the model is robust (not over-sensitive) to the hyperparameters, since small changes in these do not lead to qualitatively different responses, but to proportional changes within a qualitatively similar behavior.

The probability densities in Fig. 13 provide a visualization of the energy distribution for the latter half of the simulations. Rather than showing the system’s dynamics through time, these plots summarize the energy in the system as it reaches a stable state. By including five different hyperparameter settings (corresponding to those used to produce the plots in Figs. 10, 11 and 12), each of them simulated 50 times, we obtain enough data to generate conclusions with confidence. All the densities show that the highest frequency of occurrence is located around zero, except for the pro-tumoral energy (and consequently the TME energy) in the Escape phase. These results demonstrate how the system’s behavior is clearly captured by the energy densities as quantitative information that can be subjected to statistical analysis. For instance, examining the difference between the mean and the median values (Table 5) we can determine whether the corresponding distribution is left-skewed or right-skewed. A pro-tumoral median value of zero and a left-skewed TME distribution describe a system in the Elimination phase, while a TME median value of zero with a right-skewed distribution characterizes a system in the Equilibrium phase. A bimodal right-skewed TME distribution with a large median, signals that the system is in the Escape phase.

**Fig. 14:**
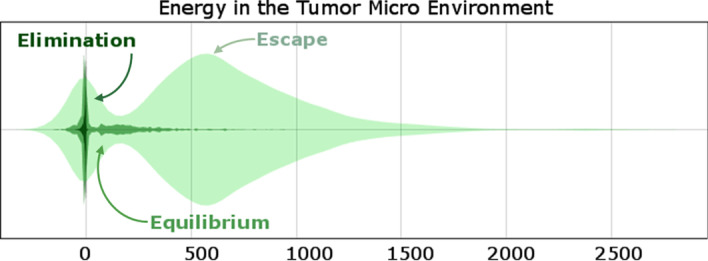
Energy probability density estimates in different phase of CI

The densities of the TME in Fig. 13 are presented in a combined form in Fig. 14. This can be interpreted as snapshots of a system’s energy as it goes through the three phases of CI. In Elimination, the median pro-tumoral energy is zero. A system in Equilibrium has a median TME energy of zero but with a right-skewed distribution that indicates that CCs are kept in check but they are not being completely eliminated. A system in the Escape phase exhibits a lot of energy, both anti-tumoral and pro-tumoral; this can be related to the presence of more CCs compared to the other CI phases and more interaction between CCs and IS elements. The much wider spread of the distribution shows that systems in the Escape phase behave much more heterogeneously than in the other phases of CI. The bimodality of the distribution in the Escape phase indicates the presence of anti-tumoral and pro-tumoral elements in the system, while the higher frequency and larger values of the positive mode identify the prevalence of the CCs in the system.

## Conclusion

By conceiving the cellular-level interaction between cancer and the immune response as a confrontation between two adversaries (each of them with multiple agents) whose actions are governed by probabilistic rules, we developed a computational model of the biological system in the TME and framed this model under the more general Ising model from statistical mechanics. This allowed us to analyze the system’s status through time by means of its Hamiltonian. Through simulation of our proposed model in a multi-agent environment, we were able to verify its operation and generate the different phases involved in Cancer Immunoediting. These results show the ability of this model to capture complex stochastic behavior in a manner that is useful to understand the system and, eventually, control its outcome. Significantly scaling up the populations of cancer cells and IS elements is a future challenge that implies very time-consuming simulations, but we foresee that the results and conclusions would remain qualitatively similar to those reported herein.

The proposed quantitative analysis method integrates variables and parameters that model some of the biochemical elements in the TME; the system’s Hamiltonian captures these in an equation that expresses how the interaction among such simple cells emerges as the Cancer-IS complex behavior. The biological system in the TME is well described by our computational model, which can be tuned to general (Elimination, Equilibrium or Escape) or specific conditions of a unique organism, given the appropriate data. The cellular interaction in CI comprises stochastic processes that are accounted for in our current model by means of flexible probabilistic rules and driven in this study by Gompertzian growth. Nevertheless, the methodological proposal is not restricted to these choices; thus one can incorporate the formalisms that best fit the laboratory data available. It is worth mentioning that to date we have observed a scarcity of real data that could be employed in the validation of CI models; this unavailability of data is attributed to the complexity of the CI process and the numerous associated obstacles for its observation in the laboratory.

The method presented herein opens alternatives for exploring different aspects of the Cancer Immunoediting process; for instance, the variables and parameters corresponding to the biochemical elements in anti-cancer therapies may be directly integrated through the Ising-model Hamiltonian formalism, such that complementary extensions of the proposed model may capture the system’s dynamics that quantify the effect of diverse (chemo-, radio- or immuno-) therapies. In future work we will pursue these ideas by combining our findings with important contributions in related studies, particularly those related to DE-based and hybrid modeling of therapeutic elements. Our ultimate goal is the development of a comprehensive energy-based model of cancer immunology.

## Supplementary Information

**Additional file 1.** Sensitivity analysis and analysis of model elements.

## Data Availability

The datasets generated and/or analyzed during the current study are not publicly available due to limitations imposed by our funding agency, but are available from the corresponding author on reasonable request.

## Abbreviations

ABM: Agent-based model
AIS: Adaptive immune system
CC: Cancer cells
CI: Cancer immunoediting
IIS: Innate immune system
IS: Immune system
M$$\upvarphi$$s: Macrophages
Ns: Neutrophils
NKs: Natural Killers
ODE: Ordinary differential equations
PDE: Partial differential equations
SDE: Stochastic differential equations
TME: Tumor micro-environment

## Footnotes

1 In computer science, an energy-based model associates a scalar probability (called energy) to each configuration of observed and latent variables; the name is imported from statistical physics to machine learning, without the literal meaning.

2 This expression is found in Additional file 1: S1 Text of the cited work.
