# Supplementary material for: Modeling cancer immunoediting in tumor microenvironment with system characterization through the ising-model Hamiltonian
Source: BMC Bioinformatics. 2022 May 30;23:200. doi: 10.1186/s12859-022-04731-w (PMC9150349; doi:10.1186/s12859-022-04731-w)
Supplement: Supplementary file 1 — Additional file 1. Sensitivity analysis and analysis of model elements. [file 12859_2022_4731_MOESM1_ESM.pdf]

Supplementary Material

Modeling Cancer Immunoediting in Tumor Microenvironment
with System Characterization through the Ising-model Hamiltonian
BMC-Bioinformatics

Alfonso Rojas-Domínguez, alfonso.rojas@gmail.com
Matías Alvarado, matias@cs.cinvestav.mx

March 14, 2022

Sensitivity Analysis

Parameter ranges corresponding to Elimination, Equilibrium and Escape phases of Cancer Immuunoediting (CI) are shown in Fig. 1 in shades of green, yellow and red, respectively. Considering that $\sigma_1 = \sigma_2$ for every simulation, each of the ranges, represented by a semi-transparent horizontal bar, corresponds to one combination of two hyperparameters (μ_1, μ_2). The color saturation illustrates the overlap between the range of the corresponding parameters. As can be observed, with the exception of the Max. Age of IS-cells, the range of values tested for all paremeters is quite large.

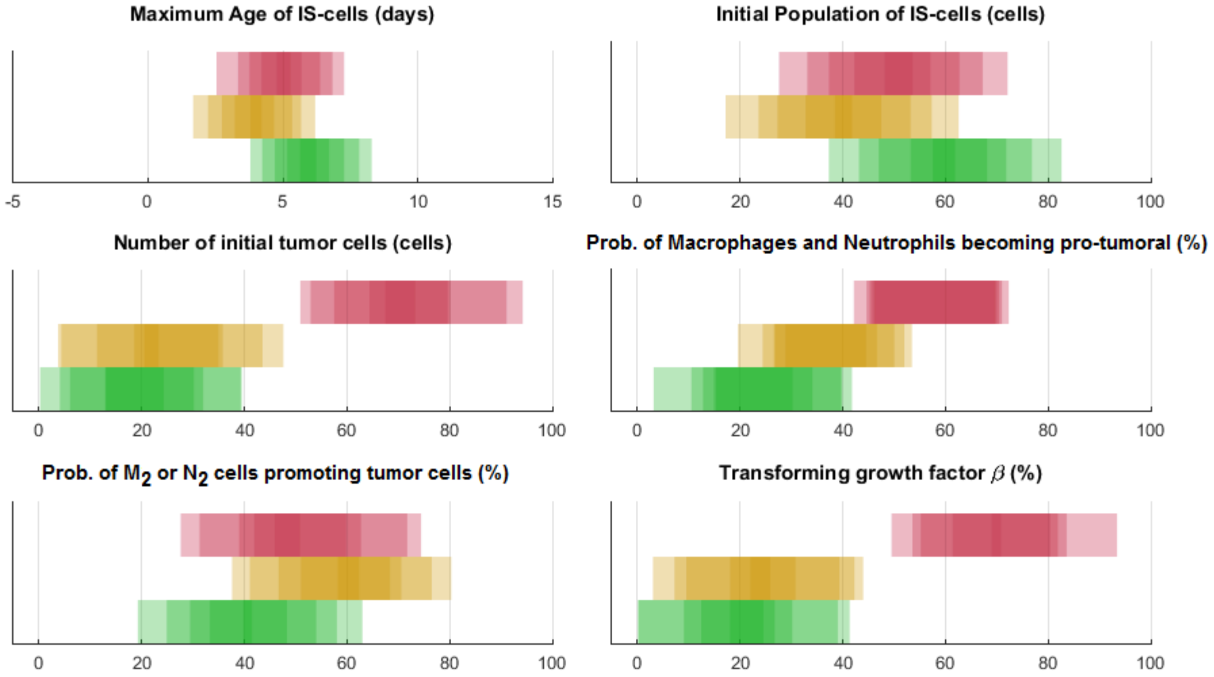

Figure 1: Representative ranges of parameter values on which the model was simulated.

The effect of each pair of hyperparameters μ_1 (anti-tumoral strength) and μ_2 (pro-tumoral strength) on the elements of the model, for each of the phases of CI, is illustrated in Fig. 2. What is measured is the average (over 100 simulations) of the median of the differences between adjacent elements of the sequences of cell amounts, over time. These differences are an approximation to the derivative, therefore what is shown is the tendency of the anti-tumoral and pro-tumoral elements of the system to increase or decrease depending on the hyperparameters. The colorbar at the bottom of Fig. 2 provides a reference between the colors used and the numerical values represented.

It can be observed that as one moves from one pair of hyperparameters to the next in sequence (i.e. along the diagonals of the matrices illustrated), their effect on the two groups of model elements changes gradually. In other words,

moderate changes of the hyperparameters result in proportionally moderate changes on the anti-tumoral and pro-tumoral populations. The second observation that can be made is that the populations of cells in general tend to decrease over time (as discussed below). The exception is the population of pro-tumoral elements in the Escape phase, whose approximate derivative exhibit positive numbers, indicating that this population increases over time, as expected to occur in this phase.

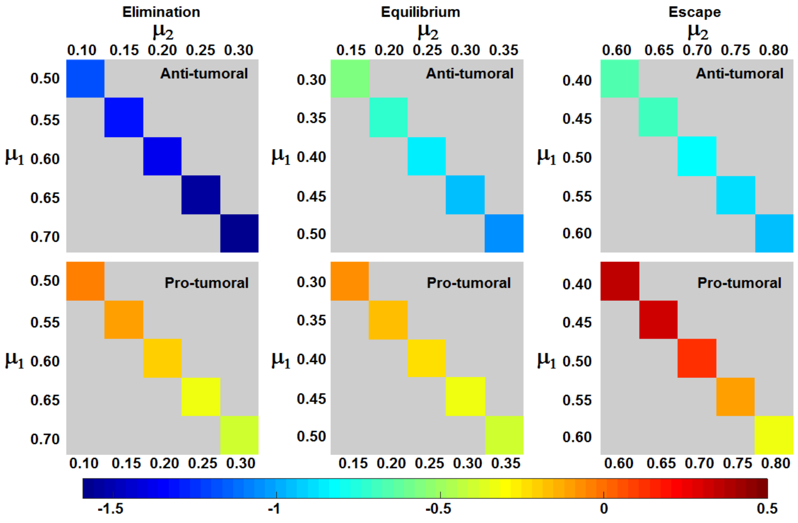

Figure 2: Sensitivity matrix for hyperparameters μ_1 and μ_2 on Anti-tumoral (top) and Pro-tumoral (bottom) agents. Colored squares represent the median of the derivative over time (avg. over 100 simulations).

To better observe the changes caused by the diffrent pairs of hyperparameters, the information in the sensitivity matrix is show as a 3D plot in Fig. 3. In this plot, instead of on a color scale, the changes are shown along the vertical axis.

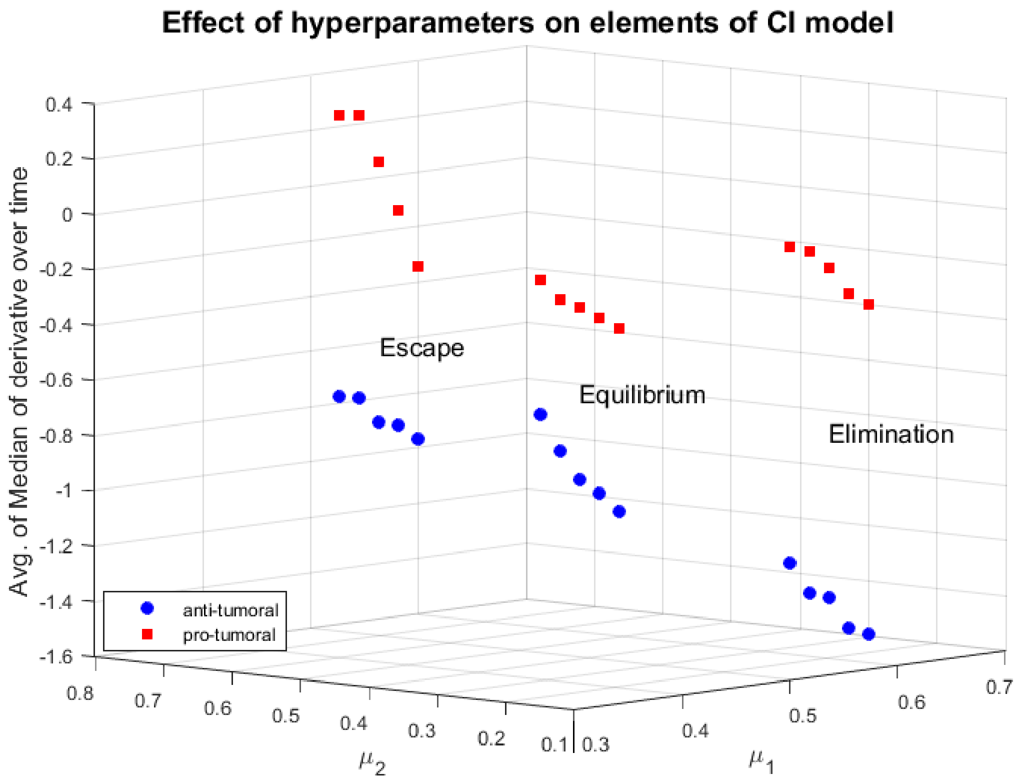

Figure 3: 3D plot of the median of the approx. derivative for each combination of hyperparameters. Each point is the average over 100 simulations.

Analysis of Model Elements

Besides the analysis of the model in terms of anti-tumoral elements and pro-tumoral elements collectively, each of the cell types in these categories is analysed independently. The behaviour of model elements over time, organized by cell-type and by phase of CI, is shown in Fig. 4.

In the Elimination phase the behaviors of Ns, NKs, Mφs and T_{reg} cells are indistinguishable from each other. Similarly, the behaviors of CD8+T and CD4+T cells are also equivalent to each other, but different from the prior elements. On the other hand, the plot of tumor cells (first row, left column) shows that in this CI phase these cells are eliminated within the first few iterations (days). This matches the simulations in [1].

In the Equilibrium phase, Ns, NKs, and Mφs cell types behave similarly to each other, but this time CD8+T, CD4+T and T_{reg} cells group together. In this phase, the tumor cells are not always eliminated over time, but in some simulations (in aprox. 50% of them) these cells survive and even increase their number. Notice as well that their plots are shown in a scale of 0 to 50, so their number is comparable to that of the IS cells as a collective. These observations are consistent with how CI has been theorized to behave in the Equilibrium phase.

In the Escape phase, Ns, NKs, and Mφs cell types again behave similarly to each other, while CD8+T, CD4+T and T_{reg} cells can be grouped together. In this phase the tumor cells start by increasing their number, by the middle of the simulation time (day 50) their amount has decreased (due to the action of the anti-tumoral cells), and from this point their number grows again. The scale required in this phase for the amount of cells is abut three times the one used in the Equilibrium phase, so the behaviors of tumor cells in these two phases are not comparable. In the Escape phase the tumor cells always survive and increase their number very significantly. This essentially corresponds with the definition of what constitutes the presence of cancer.

As discussed in our paper, the different behaviors observed in Fig. 4 are explained as emergent behavior attributed to the stochastic interaction between the multiple agents that form the system. In other words, it is important to emphasize that these different behaviors were not explicitly programmed into the system, but arise naturally from the interplay between agents that have very simple rules imbedded into them.

Another aspect which is worthy of comment is the data scatter observed for different types of cells and different CI phases. In Elimination, the largest variability is observed in the simulations of CD4+T and CD8+T cells. In Equilibrium and Escape, the largest variability is observed in the simulations of CCs, Ns, NKs, and Mφs, with the plots of the CCs in the Escape phase exhibiting the largest variability overall. Once again, these different degrees of variability, and when or for which cell types occur, were not explicitly programmed into the system.

Conclusion

This document presents supplementary material to the simulation results and analyses presented in our paper. Jointly, these materials constitute our current evidence w.r.t. to the working of our model of CI. In future work our intention is to carry out a detailed analysis of the local (in time and space) interactions between our agents, to explain the specific mechanisms that drive the qualitative behaviors observed so far.

Acknowledgment. This work was supported by the National Council of Science and Technology of Mexico (CONACYT) through grants: A1-S-20037 (M. Alvarado, lead researcher) and CÁTEDRAS-2598 (A. Rojas-Domínguez).

References

[1] Daniel K Wells, Yishan Chuang, Louis M Knapp, Dirk Brockmann, William L Kath, and Joshua N Leonard. Spatial and functional heterogeneities shape collective behavior of tumor-immune networks. *PLoS computational biology*, 11(4):e1004181, 2015.

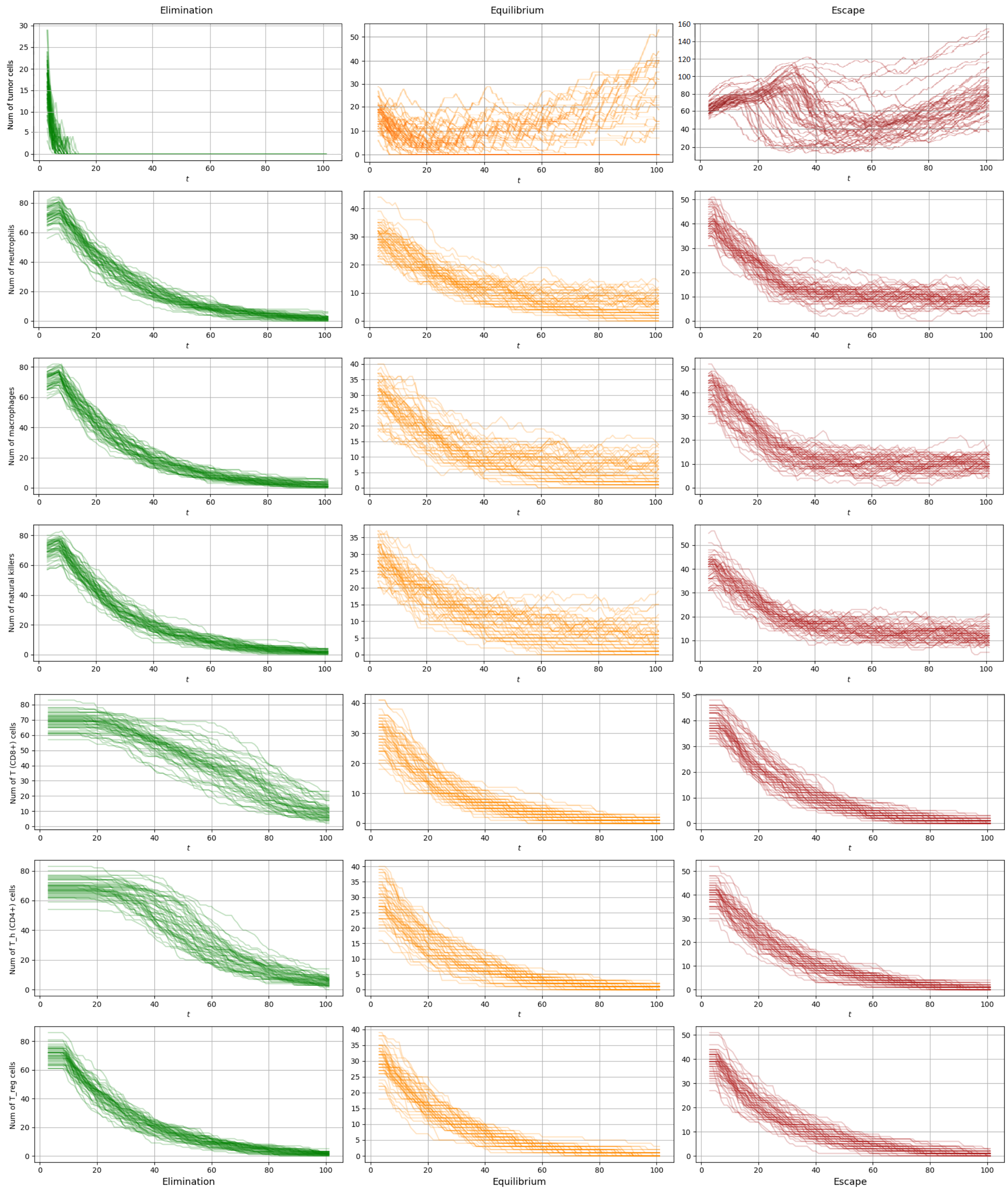

Figure 4: Model variables, time series plots, each panel represents 50 simulations. Notice the different scales for the vertical axes.
